# Supplementary material for: Gaussian process emulation for exploring complex infectious disease models
Source: PLoS Comput Biol. 2025 Dec 29;21(12):e1013849. doi: 10.1371/journal.pcbi.1013849 (PMC12774377; doi:10.1371/journal.pcbi.1013849)
Supplement: S1 Text — (PDF) [file pcbi.1013849.s001.pdf]

S1 Text. Detailed individual-based model description

This detailed individual-based model (IBM) description is motivated by previous studies using the Overview, Design concepts, and Details (ODD) protocol for describing IBMs [1–3]. The IBM was implemented in C++.

1. Purpose and patterns

The purpose of our IBM is to explore how human movement, social population structure, and seasonal variation in infection probability influence infectious disease dynamics. Rather than replicating a specific empirical system, the model is intended to highlight the relative importance of these parameters and their interactions in shaping disease spread. Its performance is evaluated by examining how the absolute number of infectious cases and the change in infectious cases over time are affected by IBM parameter changes.

2. Entities, state variables, and scales

The model operates in daily timesteps and includes two primary entities: humans and locations. Each location represents a residential home with a group of residents, who are collectively considered a 'family'. There are no distinct non-home locations such as workplaces, schools, or other public spaces. The number of humans in a given family — the number of humans residing at a given home location — is drawn from a negative binomial distribution with $\mu = 6.2$ *and* $\theta = 9.07$, following the household size distribution observed in Iquitos, Peru [4,5].

Each individual human has the following state variables: a home location, an infection status (susceptible, exposed, infected, or recovered), the number of remaining days in their current infection status (how much longer they will stay in their current state), and the number of elapsed days in their current infection status (how long they have already been in that state).

The basic spatial unit of the model is the location. Simulations are initialized by generating 10,000 locations. Each location has the following state variables: the number of infectious humans visiting it in the current timestep of the model, its per-contact infection probability (the probability that an infectious individual that visited the location at time $t-1$ transmits the disease to a susceptible individual that visits the same location at time t), a history of the number of infectious visitors it has had, and a history of its infection probability over time. All locations are randomly grouped into family clusters of a user-specified size. Each location is assigned to only one family cluster, ensuring that no locations are shared between clusters. All family

members of a single home location belong to the same family cluster, but not all members of a given family cluster reside in the same home location. S1 Fig provides a conceptual overview of family clusters (and human movement) as implemented in our IBM. Conceptually, then, a family cluster is a group of locations, the members of which tend to socialize together; an individual in a given family cluster is more likely to visit a location inside the family cluster than to visit a location outside it. This grouping introduces social structure into the simulation (please refer to the next section for further details). Note that family clusters are conceptually equivalent to the social groups defined by Reiner *et al.* (2014) [5].

3. Process overview and scheduling

The two core processes of the model are human movement and infection dynamics.

Human movement

For the process of human movement, during each timestep the model iterates through all individuals to determine which locations they will visit. Each individual visits its home location at least once every timestep. For each individual, an additional number of visits by the individual is drawn from a negative binomial distribution at the beginning of each day. The use of a negative binomial distribution allows for heavy-tailed human mobility distributions where some individuals are highly mobile, visiting a large number of locations per day. However, there is no correlation between the various sampled values for a given individual over time, meaning that we did not model systematic super-spreaders in our IBM. The infection status does not affect human movement in our IBM: infectious individuals visit, on average, the same number of locations as susceptible or recovered individuals.

For each visit, whether the visit is to a location inside the individual's family cluster or to a location outside that cluster is determined probabilistically, with the social structure parameter being the probability that a particular visit happens within the family cluster (Table 1). Locations to be visited are then randomly selected from the set of locations inside or outside the individual's family cluster, as appropriate. Multiple visits to the same location are allowed. S1 Fig provides a conceptual overview of human movement patterns as implemented in our IBM.

Infection dynamics

In our model, each human can have one of four infectious states: susceptible, exposed, infectious, and recovered. The model tracks the number of days each simulated human will remain in its current infection state, decreasing this count at the end of each day.

At the start of the simulation, all humans are susceptible. The disease is then introduced into the population by randomly selecting one individual and immediately changing their infection status from susceptible to exposed. This exposed status indicates that the individual has contracted the disease but is not yet infectious. Unless specified otherwise, exposed individuals become infectious at the end of each day, effectively reducing the model to a Susceptible-Infectious-Recovered model, where the length of the infectious period is specified by the user. At the end of an individual's infectious period, the individual's infection status changes to recovered the next day. The model assumes lasting immunity, so once individuals recover, they cannot be reinfected.

If a susceptible human visits a location that had *N* infectious visitors the day before, the probability of contracting the disease and immediately entering the exposed state is $1 - (1 - p_{infection})^N$ where $p_{infection}$ is the infection probability per contact. (This is simply the probability that a binomial draw B(*N*, $p_{infection}(t)$) ≥ 1, indicating that infection occurred from at least one previous visitor.) The infection probability $p_{infection}$ follows a cosine function that is determined by three parameters: the average infectivity (α_0), the seasonality strength (α_{season}), and the first case timing (t_{first}), and is calculated as follows:

$$p_{infection}(t) = \alpha_0 * (1 + \alpha_{season} * cos(2\pi * (t/365 - t_{first})))$$

Each location is assigned the same infection probability for a given day, as determined by the cosine function above. Variations in the overall likelihood of infection, from location to location, arise from the differing numbers of infectious individuals visiting each location.

If a visiting human is already infectious, the model increments the count of infectious visits for the current day at each location visited by that human, which will make those locations "infectious" in the following timestep as just described.

Since humans only change their infection status at the end of each day, and the likelihood of infection for susceptible individuals is determined by the number of infectious visitors from the previous day, the order in which individuals are processed is inconsequential. This ensures that the model remains asynchronous and order-independent during each day. Indeed, this design would allow the model to be parallelized to run across multiple processing cores, although runtimes were fast enough that we did not deem that necessary.

4. Design concepts

Basic principles

The model aims to study abstract disease dynamics within human populations exhibiting varying levels of social structure. It does not focus on the realistic modeling of a specific city, or on the biological details of a specific disease.

Emergence

The number of infectious individuals each day — the central output of this model, for our purposes — is an emergent property, not predefined within the model. Stochasticity plays a major role in introducing uncertainty into these patterns.

Adaptation, objectives, learning, prediction, and sensing

None of the individuals in the model have the ability to adjust their behaviors. There are no adaptive behaviors, learning abilities, predictive capabilities, or sensing capabilities in the model. Our model therefore represents a non-cognitive variant of individual- or agent-based models, sometimes also referred to as a microsimulation [6].

Interaction

Humans interact by potentially infecting other humans who visit the same location the next day.

Stochasticity

This IBM incorporates stochasticity in the family sizes, the daily number of visits per human, and the probabilistic infection dynamics. For detailed descriptions, please refer to Sections 2 and 3.

Collectives

Each human is assigned a home location and a family cluster, making them members of a family and a collection of locations. However, no special properties are attributed to sharing a home location or family cluster, except for the general tendency to interact more frequently due to the human movement rules described in Section 3.

Observations

The model outputs a table of the counts of susceptible, exposed, infectious, and recovered individuals for each day of the simulation, across the entire population.

5. Initialization

Please refer to Sections 2 and 3 regarding the initialization of the model.

6. Input

The model has eight parameters that can be specified by the user with command-line arguments (Table 1).

Out of these eight parameters, three parameters collectively influence the infection probability, as described by the equation in Section 3: the average infectivity, the seasonality strength, and the first case timing. Additionally, the user must define:

- The length of the infectious period, after which infectious individuals transition to being recovered (Section 3: *infection dynamics*).
- Parameters for the negative binomial distribution describing human movement (Section 3: *human movement*).
- The proportion of visits that occur within the family cluster of an individual (Section 3: *human movement*).
- The number of locations per family cluster (Section 2).

References

1. Grimm V, Berger U, Bastiansen F, Eliassen S, Ginot V, Giske J, et al. A standard protocol for describing individual-based and agent-based models. Ecol Modell. 2006;198: 115–126. doi:10.1016/j.ecolmodel.2006.04.023

2. Grimm V, Railsback SF, Vincenot CE, Berger U, Gallagher C, DeAngelis DL, et al. The ODD protocol for describing agent-based and other simulation models: A second update to improve clarity, replication, and structural realism. J Artif Soc Soc Simul. 2020;23: 7. doi:10.18564/jasss.4259

3. Perkins TA, Reiner RC Jr, España G, ten Bosch QA, Verma A, Liebman KA, et al. An agent-based model of dengue virus transmission shows how uncertainty about breakthrough infections influences vaccination impact projections. PLoS Comput Biol. 2019;15: e1006710. doi:10.1371/journal.pcbi.1006710

4. Stoddard ST, Forshey BM, Morrison AC, Paz-Soldan VA, Vazquez-Prokopec GM, Astete H, et al. House-to-house human movement drives dengue virus transmission. Proc Natl Acad Sci U S A. 2013;110: 994–999. doi:10.1073/pnas.1213349110

5. Reiner RC Jr, Stoddard ST, Scott TW. Socially structured human movement shapes dengue transmission despite the diffusive effect of mosquito dispersal. Epidemics. 2014;6: 30–36. doi:10.1016/j.epidem.2013.12.003

6. Orcutt GH. A New Type of Socio-Economic System. Rev Econ Stat. 1957;39: 116. doi:10.2307/1928528
