# Supplementary material for: Gaussian process emulation for exploring complex infectious disease models
Source: PLoS Comput Biol. 2025 Dec 29;21(12):e1013849. doi: 10.1371/journal.pcbi.1013849 (PMC12774377; doi:10.1371/journal.pcbi.1013849)
Supplement: S2 Text — (PDF) [file pcbi.1013849.s002.pdf]

S2 Text. Detailed empirical data processing description

We retrieved weekly dengue incidence data at the municipality level for Colombia from the OpenDengue database, an open-access platform that provides detailed epidemiological data on dengue [1]. The selected dataset spans from January 1st, 2007, when weekly resolution data became consistently available, to December 31st, 2019, comprising 163,279 entries. We chose to end in 2019 to avoid the potential confounding effects of the COVID-19 pandemic [2]. We chose Colombia for this study because it is one of the countries most affected by dengue in the Americas [3] and offers exceptionally well-documented time series data on dengue incidence [1]. To adjust dengue incidences for potential under-reporting and asymptomatic cases, reported dengue incidences were corrected by a factor of 25 [3,4]. Estimates for under-reporting factors in dengue typically range from 10 to 27, depending on the region and study [4]. While we recognize that using a correction factor of 25 for Colombia, which has well-documented dengue incidence records [1], might be cautious, we chose it to include a broad range of municipalities.

We obtained municipality-level processed data from Siraj *et al.* (2018) [5], which provides a global, high-resolution dataset of potential environmental drivers for Zika transmission in Colombia between January 1st, 2014 and October 1st, 2016. Although the data published by Siraj *et al.* (2018) focused on Zika, it is relevant to dengue because both viruses share a primary vector, *Ae. aegypti*, which is responsible for the majority of dengue transmission in Colombia [6]. Specifically, we used four metrics from Siraj *et. al* (2018) (i) the population count (ii) the weekly occurrence probabilities of *Ae. aegypti* [7] (iii) the Gross Cell Product, which measures economic activity at a fine spatial scale [8], and (iv) the mean travel time to the nearest city. Please refer to Table 1 in Siraj *et al.* (2018) for additional information on the municipality-specific data.

We matched the records from Siraj *et al.* (2018) and Clarke *et al.* (2024) based on the names of municipalities and their respective departments. To improve the matching, we standardized the municipality and department names by converting them to lowercase and applying a latin-ascii transformation to remove any accents or special characters. In cases where mismatches occurred, either at the municipality or departmental level, we followed a similar approach to Clarke *et al.* (2024), manually reviewing the records and checking the geographic boundaries using shapefiles. While we were able to obtain the original shapefiles from Clarke *et al.* (2024) (Oliver Brady, personal communication), the original shapefiles for Siraj *et al.* (2018) were not accessible at the time of our study. As a substitute, we used shapefiles from the OCHA

database [9]. Despite this limitation, we successfully matched 95% (1,009 out of 1,063) of the municipalities present in the raw dengue incidence data. Our goal was not to achieve a perfect match, but rather to secure a sufficient number of high-quality matches to proceed with our analysis.

We focused on 211 municipalities that aligned with our individual-based model in terms of population size (i.e., at least 30,000 individuals) and had a maximum dengue incidence rate of at least 0.1% over the entire study period. To detect epidemics, we fitted a smoothing spline using the ss() function from the npreg R-package ($\lambda = 10^{-10}$) [10]. An epidemic outbreak was defined as a period of at least four consecutive weeks in which the spline function exceeded the median dengue incidence rate. Using this method, we identified 1,211 potential epidemic outbreaks with an $i_{\max}$ of at least 0.1% which were included in the analysis. On average, each municipality had 6.34 outbreaks. The average duration per outbreak was 195 days, with an average $i_{\max}$ of 0.6%.

References

1. Clarke J, Lim A, Gupte P, Pigott DM, van Panhuis WG, Brady OJ. A global dataset of publicly available dengue case count data. Sci Data. 2024;11: 296. doi:10.1038/s41597-024-03120-7

2. Cavany SM, España G, Vazquez-Prokopec GM, Scott TW, Perkins TA. Pandemic-associated mobility restrictions could cause increases in dengue virus transmission. PLoS Negl Trop Dis. 2021;15: e0009603. doi:10.1371/journal.pntd.0009603

3. Gutierrez-Barbosa H, Medina-Moreno S, Zapata JC, Chua JV. Dengue infections in Colombia: Epidemiological trends of a hyperendemic country. Trop Med Infect Dis. 2020;5: 156. doi:10.3390/tropicalmed5040156

4. Bhatt S, Gething PW, Brady OJ, Messina JP, Farlow AW, Moyes CL, et al. The global distribution and burden of dengue. Nature. 2013;496: 504–507. doi:10.1038/nature12060

5. Siraj AS, Rodriguez-Barraquer I, Barker CM, Tejedor-Garavito N, Harding D, Lorton C, et al. Spatiotemporal incidence of Zika and associated environmental drivers for the 2015-2016 epidemic in Colombia. Sci Data. 2018;5: 180073. doi:10.1038/sdata.2018.73

6. Mejía-Jurado E, Echeverry-Cárdenas E, Aguirre-Obando OA. Potential current and future distribution for Aedes aegypti and Aedes albopictus in Colombia: important disease vectors. Biol Invasions. 2024;26: 2119–2137. doi:10.1007/s10530-024-03298-2

7. Kraemer MU, Sinka ME, Duda KA, Mylne AQN, Shearer FM, Barker CM, et al. The global distribution of the arbovirus vectors Aedes aegypti and Ae. albopictus. Elife. 2015;4: e08347. doi:10.7554/eLife.08347

8. Nordhaus WD. Geography and macroeconomics: new data and new findings. Proc Natl Acad Sci U S A. 2006;103: 3510–3517. doi:10.1073/pnas.0509842103

9. United Nations Office For The Coordination of Humanitarian Affairs. Colombia - Subnational Administrative Boundaries. 20 Apr 2020 [accessed 22 Feb 2024]. Available: https://data.humdata.org/dataset/cod-ab-col

10. Helwig NE. Multiple and Generalized Nonparametric Regression. London: SAGE Publications, Inc.; 2020.
