# Supplementary figures and images for: Gaussian process emulation for exploring complex infectious disease models
Source: PLoS Comput Biol. 2025 Dec 29;21(12):e1013849. doi: 10.1371/journal.pcbi.1013849 (PMC12774377; doi:10.1371/journal.pcbi.1013849)

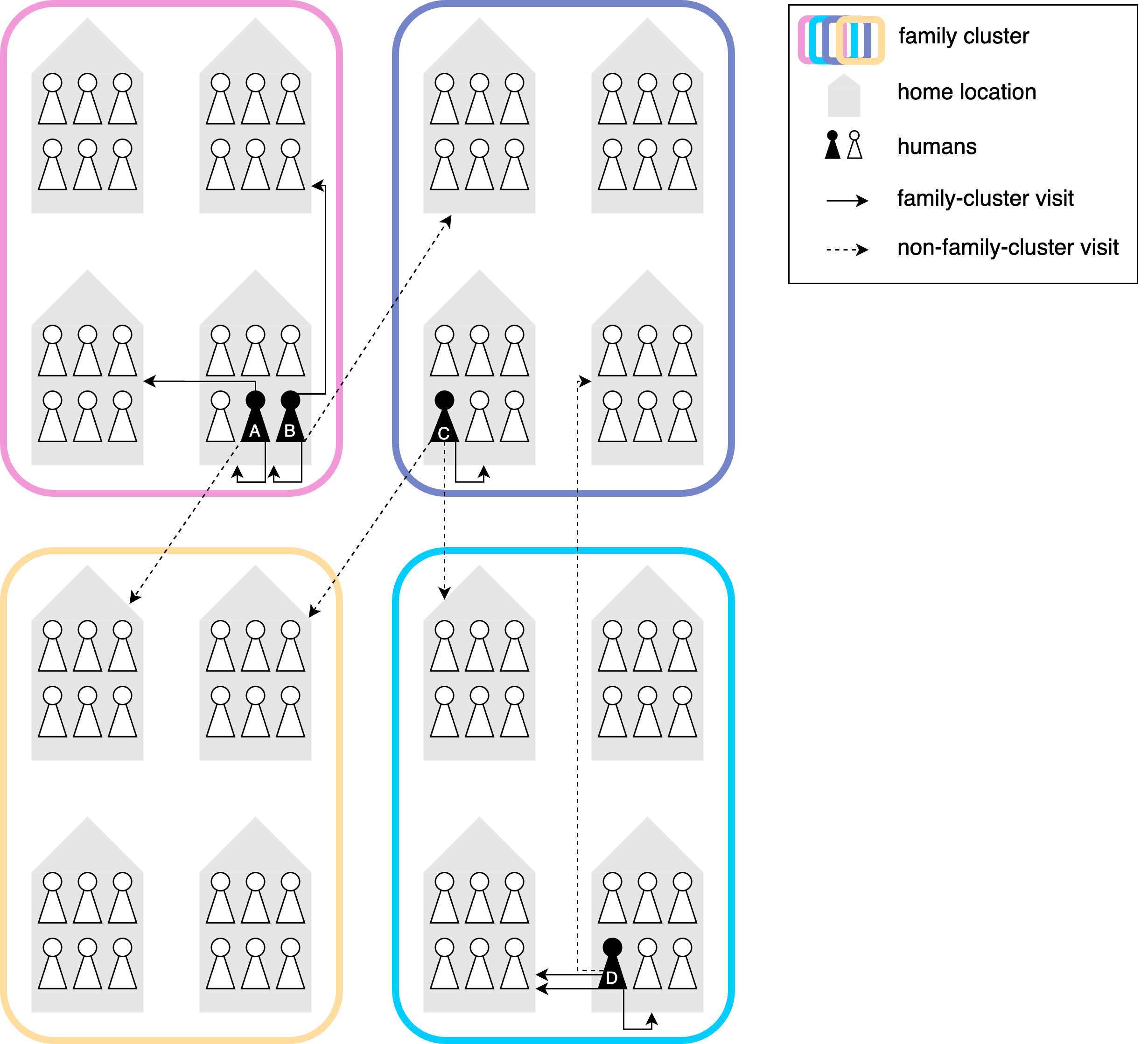

Supplement: S1 Fig — Each colored frame represents a unique, non-overlapping family cluster, with each cluster containing multiple family homes. Individuals can make visits within their own family cluster (solid arrows) or to other clusters (dashed arrows). The likelihood of visits occurring inside the family cluster is determined by the social structure parameter (Table 1). Each individual visits their home at least once per day and moves independently of others in the same family (individuals A and B). Multiple visits to the same location are allowed (individual D). Visits to other family clusters occur randomly and are not restricted to any specific cluster (individual C). (PNG) [file pcbi.1013849.s003.png]

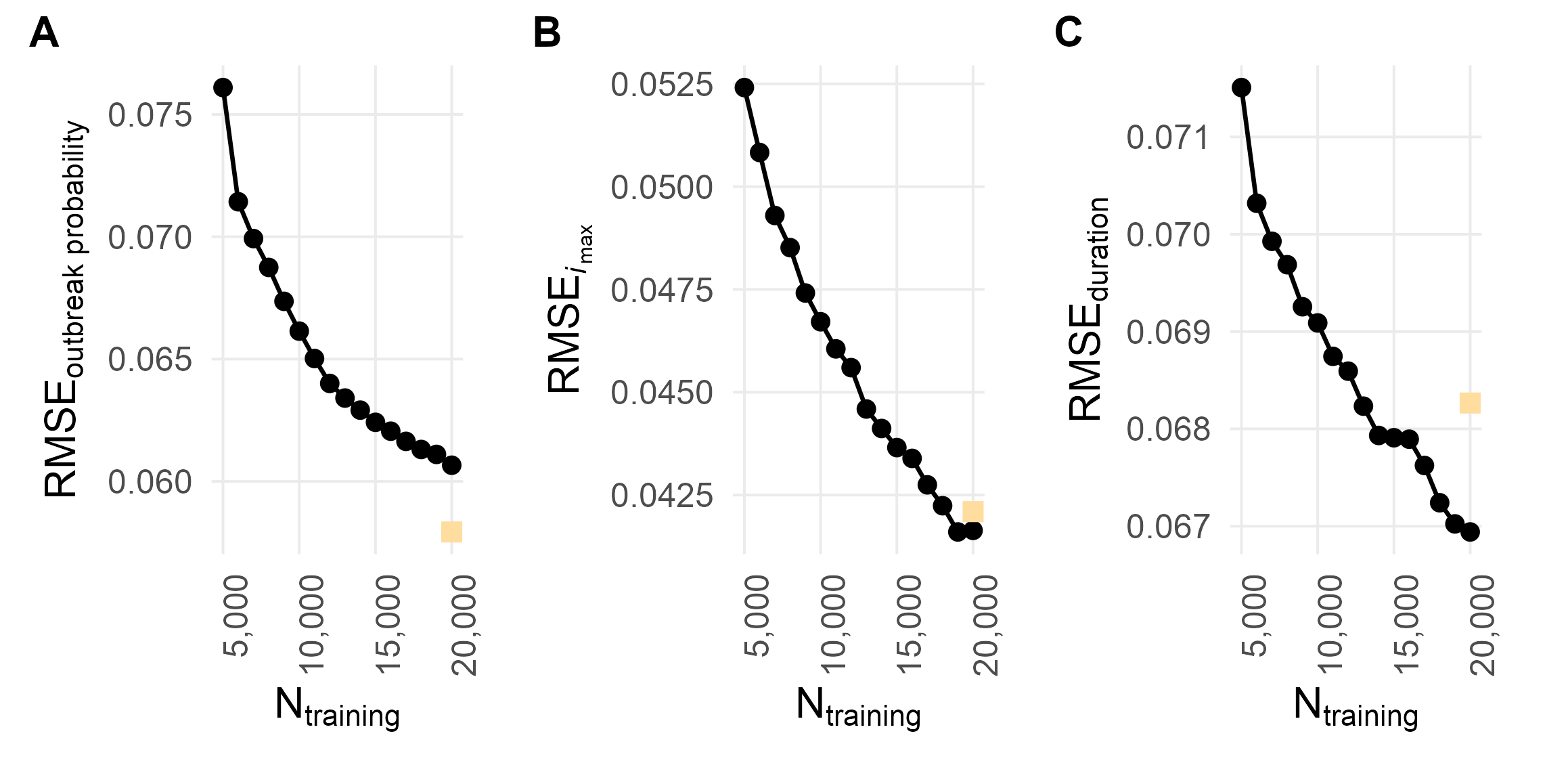

Supplement: S2 Fig — The Root mean squared error (RMSE) decreases as the size of the dataset used to train the Gaussian Processes increases (x-axis). The RMSE between the predictions of the final GP model and the test data (N = 10,000 data points) is indicated by a yellow square. (A) outbreak probability (B) maximum incidence (imax), (C) log10-transformed duration. (PNG) [file pcbi.1013849.s004.png]

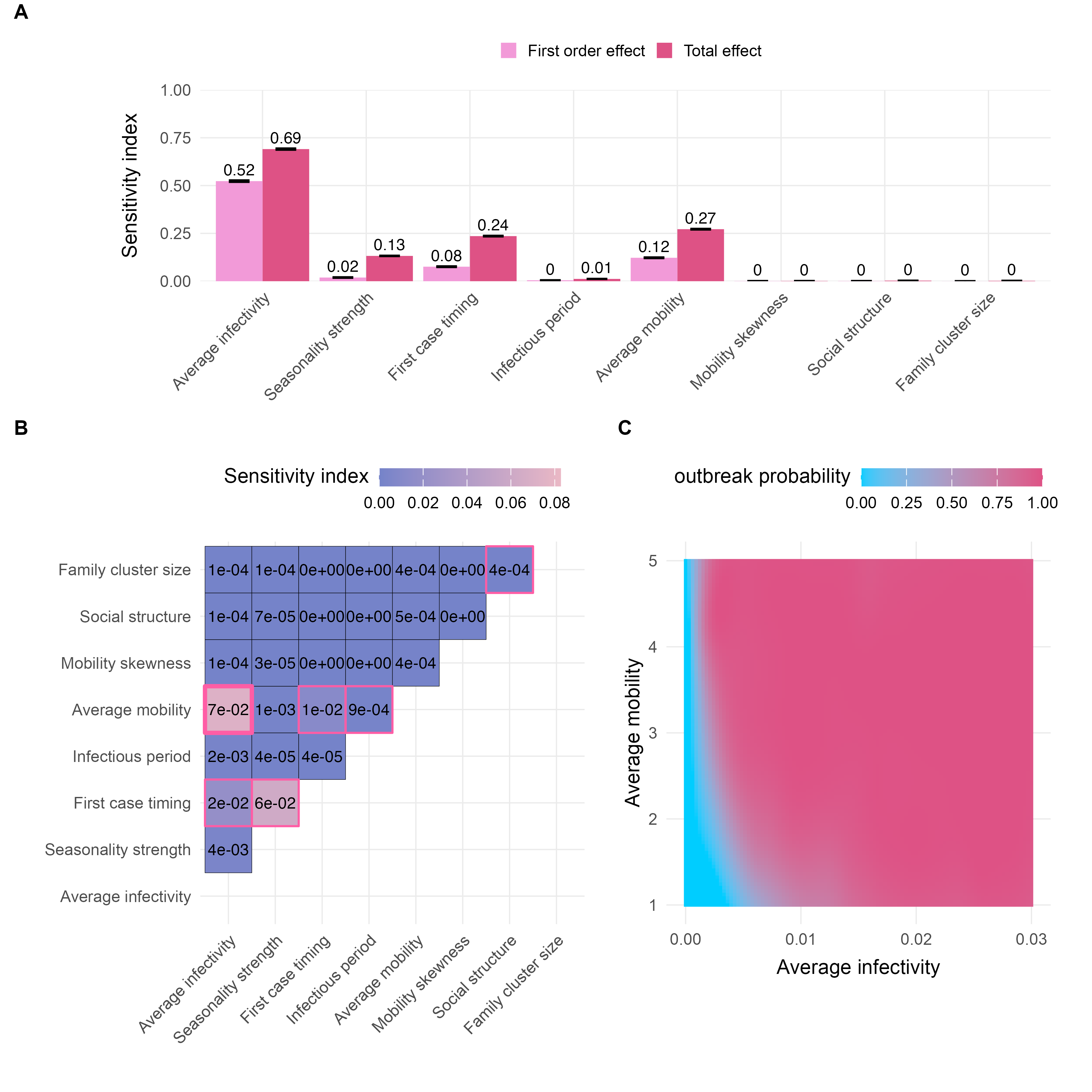

Supplement: S3 Fig — (A) First-order and total effects across the entire input domain (Table 1). The first-order effect describes the impact of a single parameter on the model output (outbreak probability), while the total effect accounts for all interactions involving one or more parameters. Error bars represent the 95% confidence intervals of the sensitivity index estimates. We evaluated a total of 9,437,184 points for the sensitivity analysis. (B) Second-order effects across the entire input domain (Table 1). A second-order effect captures the pairwise interaction between two parameters. Sobol indices with a 95% confidence interval that does not overlap zero are highlighted with a pink border. The largest second-order effect is emphasized with a bold pink border. (C) Predicted outbreak probabilities with varying average infectivity and average mobility parameters (i.e., the two parameters with the largest second-order effect, see panel B). Other parameters were fixed at default values (Table 1). (PNG) [file pcbi.1013849.s005.png]

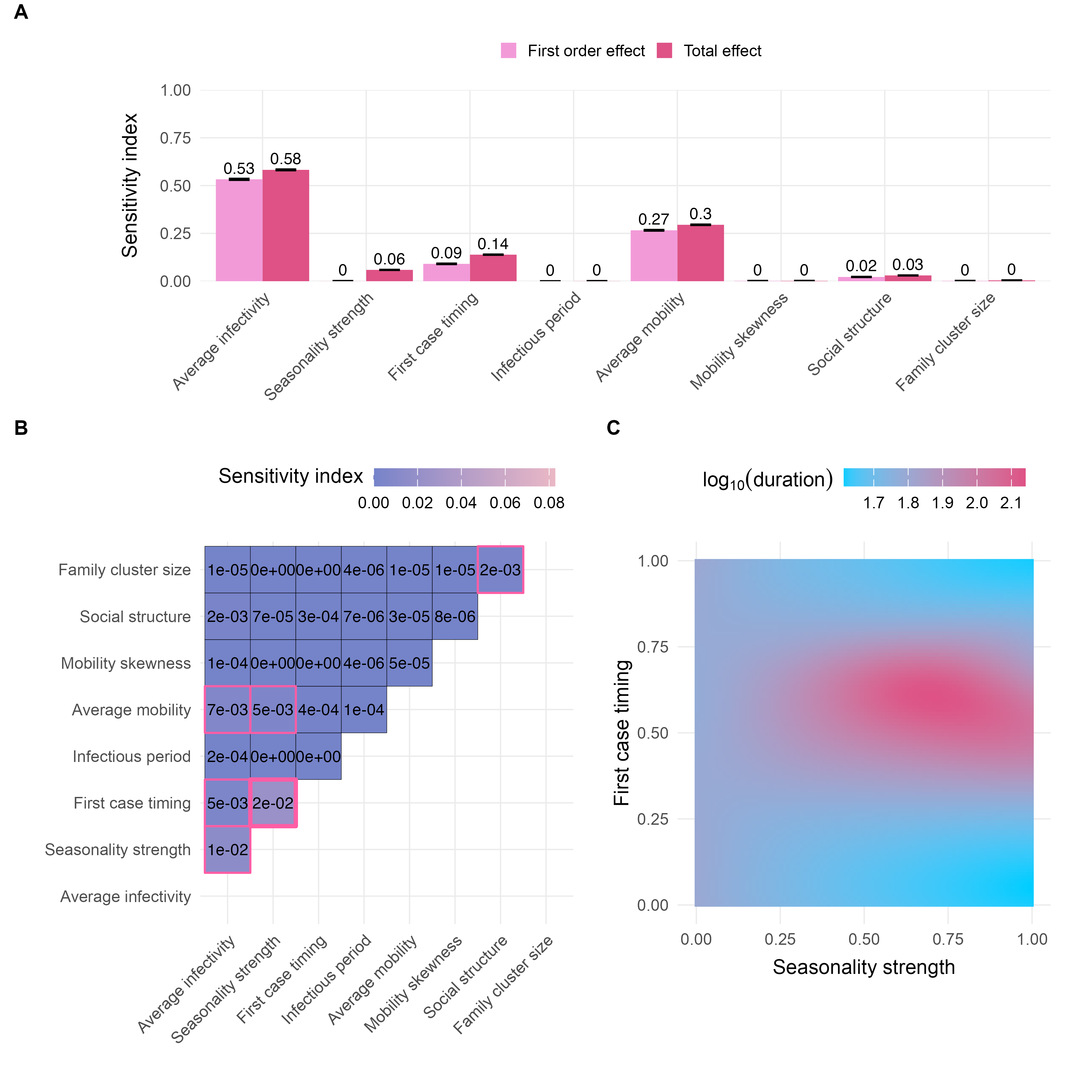

Supplement: S4 Fig — (A) First-order and total effects across the entire input domain (Table 1). The first-order effect describes the impact of a single parameter on the model output (log10(duration)), while the total effect accounts for all interactions involving one or more parameters. Error bars represent the 95% confidence intervals of the sensitivity index estimates. We evaluated a total of 9,437,184 points for the sensitivity analysis. (B) Second-order effects across the entire input domain (Table 1). A second-order effect captures the pairwise interaction between two parameters. Sobol indices with a 95% confidence interval that does not overlap zero are highlighted with a pink border. The largest second-order effect is emphasized with a bold pink border. (C) log10(duration) predictions with varying seasonality strength and first case timing parameters (i.e., the two parameters with the largest second-order effect, see panel B). Other parameters were fixed at default values (Table 1). (PNG) [file pcbi.1013849.s006.png]

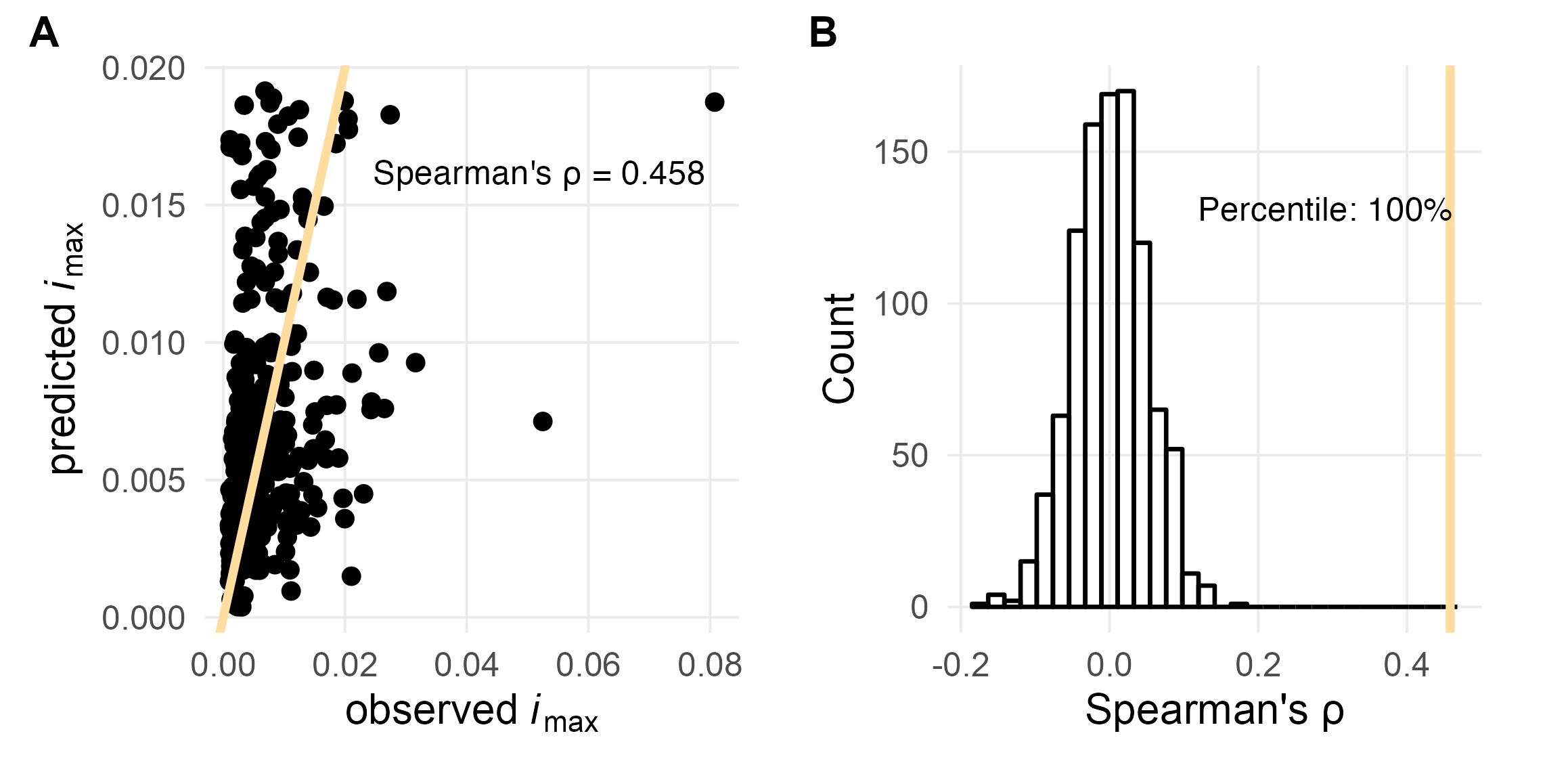

Supplement: S5 Fig — (A) Observed vs. predicted maximum incidence (imax) for empirical epidemic outbreaks (N = 449). The yellow line represents the identity line (x = y). (B) Distribution of Spearman correlation coefficients between observed and predicted imax from 1,000 permutations, where both the onset and municipality of the 449 epidemics were randomized. The actual observed correlation coefficient is shown as a vertical yellow line. (PNG) [file pcbi.1013849.s007.png]
