# Supplementary material for: Gaussian process emulation for exploring complex infectious disease models
Source: PLoS Comput Biol. 2025 Dec 29;21(12):e1013849. doi: 10.1371/journal.pcbi.1013849 (PMC12774377; doi:10.1371/journal.pcbi.1013849)
Supplement: S1 Table — Summary statistics describing the 250 parameter combinations with the lowest root mean squared errors from the parameter exploration with the Gaussian Process. These combinations represent the best-fitting sets of parameters for matching observed and predicted dengue maximum incidences across municipalities. IQR = Interquartile range (range between the 25th and 75th percentiles). (PDF) [file pcbi.1013849.s008.pdf]

# Supplementary tables

**S1 Table. Summary of 250 parameter sets with lowest RMSE from parameter exploration with Gaussian Process.**

| **Parameter** | **Min** | **Mean** | **Max** | **IQR** |
|---|---|---|---|---|
| Seasonality strength | 6e-5 | 0.05 | 0.21 | 0.05 |
| First case timing | 0.00 | 0.50 | 1.00 | 0.32 |
| Infectious period | 4.00 | 5.02 | 6.00 | 0.97 |
| Average mobility | 1.00 | 2.51 | 5.00 | 1.67 |
| Mobility skewness | 0.05 | 0.48 | 0.95 | 0.40 |
| Social structure | 0.02 | 0.63 | 0.99 | 0.53 |
| Family cluster size | 1.16 | 11.17 | 19.90 | 8.71 |
| Scaling factor | 0.01 | 0.03 | 0.10 | 0.01 |
