# Supplementary material for: Gaussian process emulation for exploring complex infectious disease models
Source: PLoS Comput Biol. 2025 Dec 29;21(12):e1013849. doi: 10.1371/journal.pcbi.1013849 (PMC12774377; doi:10.1371/journal.pcbi.1013849)
Supplement: Attachment — Submitted filename: DD-R1-response-to-reviewers.docx [file pcbi.1013849.s010.docx]

***Response to Reviewers***

***Reviewer #1:*** *The authors present a well-written paper demonstrating the use of Gaussian Process emulators for exploring the parameter spaces of epidemiological models. As a frequent user of GPs for my own ABM-centric work, I'm pleased to see another demonstration of the power and utility of this approach for the exploration of complex models; however, it's worth noting that other machine-learning methods can provide better fits for complex ABMs than GPs (see, e.g., Angione, Claudio, Silverman, Eric and Yaneske, Elisabeth (2022). Using machine learning as a surrogate model for agent-based simulations. PLoS ONE, 17(2), e0263150. (doi: 10.1371/journal.pone.0263150).*

We thank the reviewer for their positive feedback and for the helpful comment regarding alternative machine-learning methods for building surrogate models, as highlighted in Angione *et al.* (2022). We acknowledge this point in the *Introduction* section of our revised manuscript and have extended the *Discussion* section to emphasize that, besides GPs, there are several alternative emulation techniques available which may, in some cases, be preferable. For further details and the corresponding revisions in the manuscript, please refer to our point-by-point responses below.

*I find the authors' use of individual-based models (IBMs) as an overarching category to be somewhat confusing. In my career as a complex systems scientist focussed on ABMs, a distinction is normally drawn between IBMs that use transition probabilities to determine changes in agent state (e.g., microsimulations), and those that use decision rules (ABMs), with the latter being considered significantly harder to analyse. I believe the introduction to the paper would benefit from expressing more clearly the differences between individual-based and agent-based approaches, particularly for those audiences in complex systems disciplines where IBM is not a commonly used term.*

We agree that our terminology should be unambiguous to readers from various scientific backgrounds. In our primary fields of expertise (ecology, evolutionary biology, computational biology, and epidemiology), the terms individual-based model and agent-based model are often used interchangeably to describe models that simulate discrete individuals and their (inter)actions (e.g., Grimm *et al*. 2006, *Ecol. Model*).

To avoid confusion, we have revised the Introduction to use the more general term simulation model when first describing our modeling/emulation approach. We now introduce and define the term “individual-based model” (IBM) explicitly in the Methods section, where we clarify that we follow the epidemiological convention of using IBM as an umbrella term encompassing both individual- and agent-based approaches.

The revised text in the Methods section now reads:

“We implemented an individual-based model (IBM; in epidemiology, the terms "individual-based” and “agent-based” are used largely interchangeably to describe models that simulate individual entities and their interactions [2]; following this convention, we will use “individual-based model” as a general term for such modeling approaches) in C++ that simulates and tracks disease transmission and includes several parameters related to  infection probability, human movement, and social structure — three key features shaping dengue epidemics [38–40].

In our model, each individual is explicitly simulated, and each individual follows probabilistic and structural rules regarding infection and movement (though they lack adaptive, anticipatory, or learning behaviors). The model is designed not to replicate a specific empirical system, but to …”

*Other than that, the benefits of using GPs and exploring a model's parameter space more generally are explained well and with sufficient detail. I would only add here that other methods of emulating complex models can provide better emulator fit, assuming appropriate computing hardware is available; for example, neural networks are excellent at replicating models with highly non-linear behaviour, but benefit from GPUs in order to provide sufficient time savings to make the method worth the effort.*

We thank the reviewer for this helpful comment. Gaussian Processes (GPs) are a well-established emulation technique, but there are certainly other machine learning approaches (random forests, neural networks, … ) that can also be used to build effective surrogate models.

To address this point, we now briefly mention alternative machine-learning-based emulation techniques in the revised Introduction section and clarify that GPs are a commonly used approach in this context. Additionally, we have expanded the Discussion section to include a new paragraph acknowledging that neural networks can be a more suitable choice when the IBM exhibits highly nonlinear or erratic output behavior. The added paragraph reads:

“Finally, we would like to point out that GPs are only one of numerous possible choices for a surrogate model. Alternative machine learning approaches — such as random forests, support vector machines, and neural networks — can also be used to build effective surrogate models for complex IBMs. Neural networks, in particular, are well suited for capturing highly nonlinear or erratic model behavior, especially when modern computational resources such as GPUs are available [65]. We chose GPs in this study because they are a well-established emulation technique that provides a solid probabilistic foundation allowing uncertainty quantification [14]. This uncertainty quantification, in turn, enables efficient active learning strategies for selecting additional training data. Implementing such active learning strategies is considerably more straightforward with GPs than with neural networks, for which obtaining reliable uncertainty estimates is more challenging. Nevertheless, previous work has shown that neural networks can outperform GPs in predictive accuracy for highly nonlinear systems [13]. For applied emulation tasks, researchers must therefore choose the method that best fits their simulation model’s characteristics and the available computational resources.”

*The IBM presented here I would class as a microsimulation-style simulation, as the movements of the agents and the disease dynamics are apparently generated using parameters drawn from distributions rather than decision rules driving the individuals. In other words, there is top-down imposition of parameters here, which precludes the model from being an ABM, where decision rules and agent heterogeneity are used to drive population-level patterns from the bottom up. That being the case, I hope the authors can make more clear where their model sits on the IBM-ABM spectrum.*

We agree with the reviewer that our model can be classified as a microsimulation-style simulation, because movement and infection dynamics are determined by probabilistic and structural rules rather than by decision-based or adaptive behaviors of individual agents. As noted in our response above, the convention in epidemiology is to use the terms “agent-based” and “individual-based” models largely interchangeably. To clarify the position of our model for readers from different disciplinary backgrounds, we have revised the opening paragraph of the “Individual-based model” Methods section to state explicitly that our simulated individuals follow probabilistic movement rules without adaptive or learning capabilities (Please also see the quoted revised text in our manuscript provided in response to Comment “ABM/IBM terminology.”). Additionally, we highlight the parallels between our modeling approach and microsimulations in the detailed simulation model description, now provided as Supplementary Text S2.

*The results are well-written and well-presented. I'm somewhat surprised that the GPs took quite so long to run for such a simple model; my own experiences with this approach has pegged GPs as being among the fastest available emulation techniques, typically finishing ~40-50K emulated runs in a few minutes, without the use of GPUs. That being said, I'm pleased that the authors have made the effort to develop and share their own tools, which is always of benefit to the wider research community.*

We thank the reviewer for this positive comment. We agree that GP emulation is typically very fast. After our GP model was trained, emulation took about 1 second for 100,000 data points. The longer runtimes we reported refer to the time required for the initial training of the GP, which involves gradient-based methods, which can be computationally intensive depending on the dataset size, kernel choice, and number of hyperparameters. In our case, GP training took between 15 minutes and 2 hours and was positively correlated with training data size. In our revised manuscript, we explicitly separate GP training times (now in the “GP training” Methods section) from GP emulation times (now in the “GP Performance” Results section). We hope this clarifies that while GP training can be computationally somewhat demanding, the emulation step itself is indeed very fast.

*However, the GitHub archives provided need some additional work before publication. Only minimal documentation is provided for the GP Jupyter notebooks, and no readme is provided at all for the DengueSim itself. Prior to publication, I'd ask the authors to ensure that these two repositories are documented for maximum accessibility, including providing installation/running instructions and links to related literature and tutorials for beginners to these kinds of modelling techniques.*

We have thoroughly revised both GitHub repositories to improve documentation and accessibility. For the DengueSim repository (<https://github.com/AnnaMariaL/DengueSim>), we have added a detailed README file that includes compilation instructions, a brief conceptual overview of the individual-based model, examples demonstrating how to run the simulations, explanations of the model outputs, and a description of all command-line arguments with direct links to the terminology used in the manuscript.

For the DengueSim-GP repository (<https://github.com/AnnaMariaL/DengueSim-GP>), we now provide instructions for setting up the required conda environment and a comprehensive Jupyter notebook explaining how to run the GP analyses presented in the manuscript. The documentation clearly links variables and parameters to the manuscript terminology, includes READMEs describing the structure and meaning of datasets, and offers links to resources and tutorials on Gaussian Process modelling.

These updates ensure that the repositories are accessible to users with different levels of experience in individual-based and Gaussian Process modelling.

*Overall, I would say that the paper is important for the epidemiological community, where there has been some resistance to the use of complex systems methods, and demonstrating that tools exist to ease the 'curse of dimensionality' problem and provide useful analyses of their outputs will be helpful in driving the community to explore these methods more confidently. Having said that, very similar papers to this exist in other fields (demography, public health, etc.), but given the general lack of interdisciplinary communication regarding methodology I believe this paper is still valuable for its intended readership. Therefore, if the clarifications and additions suggested above are implemented, I would suggest the editors approve this paper for publication.*

We appreciate the reviewer’s recognition of the paper’s relevance to the epidemiological community.

***Reviewer #2:*** *Although I think the methods developed in the article are worth exploring, there are issues with the writing of this manuscript. Every section is too long, which makes the manuscript hard to follow. The results section starts with what is described as a 'brief' description of the results, but is about three pages long. Most of the results section is a mixture of methods and results. Much of the current methods section could be supplementary material. Information on the Gaussian processes should be more prominent.*

We thank the reviewer for the constructive feedback. To improve the readability and organization of the manuscript, we have substantially revised its structure. We now use the *PLOS Computational Biology* option to present the Methods section before the Results. This reorganization allowed us to remove the redundant method summaries that previously appeared at the beginning of the Results subsections.

We also moved the detailed description of the individual-based model (IBM) and the empirical data processing procedures to the Supporting Information (S1 Text, S2 Text). The previous “brief descriptions” of the IBM and the empirical data processing are now integrated into the Methods section. Consequently, the Results section of our revised manuscript begins directly with the Gaussian Process (GP) performance analysis, increasing the prominence of this material as suggested.

Overall, these revisions shortened the main text by approximately 18% (7 pages). We believe these changes substantially improved the manuscript’s readability, and we hope they fully address the reviewer’s concerns.

*The article advertises itself as the first paper to use gaussian process emulation to conduct sensitivity analysis. However, Golumbeanu et al [28] is referenced and also uses these methods with Sobol's sensitivity analysis.*

We agree that previous work, including Golumbeanu *et al.*, has applied GP emulation in combination with variance-based sensitivity analysis to epidemiological models. Our intention was not to claim novelty in being the first to use these methods, but rather to illustrate their application within a general framework that is largely independent of a specific disease system or highly complex simulation framework. To clarify this, we have revised the introduction to explicitly acknowledge previous studies using GP-based sensitivity analysis and to more clearly position our contribution. The revised section now reads:

“In this study, we build upon prior applications of Gaussian Process (GP) emulation for sensitivity analysis in epidemiological contexts (e.g., [32,33]) by constructing a general framework that illustrates their applicability and practical benefits. Specifically, we developed an abstract, generalizable simulation model designed to showcase how GP emulation can efficiently support variance-based sensitivity analysis across high-dimensional parameter spaces. This work illustrates the broader methodological potential of GP emulation for accelerating and interpreting complex epidemiological simulations. Our disease transmission model, which …”

***Reviewer #3:*** *This paper showcases Gaussian Process (GP) emulation as a way to make sensitivity analysis and systematic exploration tractable for complex, high-dimensional and computationally intensive individual-based models (IBMs): two tasks that would otherwise be practically impossible.

The authors trained GPs as surrogate models on input–output data from the IBM to predict three key outcomes: outbreak probability, peak incidence, and epidemic duration.

While the GPs can efficiently predict IBM outcomes and enable comprehensive sensitivity analysis, their predictive power was limited when applied to real dengue data from Colombia, most likely because the underlying IBM was highly simplified. Still, the approach identified municipalities with consistently higher infectivity estimates that overlapped with known dengue clusters, demonstrating value for capturing broad epidemiological patterns.

In my opinion, the main interest of this work lies in its methodological approach, which is clearly described and serves as a valuable proof-of-concept and a useful reference for applying Gaussian Process emulation to other, more complex IBMs. As the authors note, similar uses of GPs have already been applied to other disease specific IBMs, but their work demonstrates how the method can be applied more broadly to other existing, complex IBMs.*

We thank the reviewer for their positive and thoughtful feedback. We are pleased that they recognize the methodological contribution of our work, particularly the use of Gaussian Process emulation to make sensitivity analysis and systematic exploration of complex individual-based models more feasible. In line with this focus, and following Reviewer #2’s suggestions, we have made the Gaussian Process component even more prominent by moving the detailed individual-based model description and empirical data processing to the Supplementary Material.
